# Deep Learning Classification of Lake Zooplankton

**DOI:** 10.3389/fmicb.2021.746297

**Published:** 2021-11-15

**Authors:** Sreenath P. Kyathanahally, Thomas Hardeman, Ewa Merz, Thea Bulas, Marta Reyes, Peter Isles, Francesco Pomati, Marco Baity-Jesi

**Affiliations:** Eawag, Dübendorf, Switzerland

**Keywords:** plankton camera, deep learning, plankton classification, transfer learning, Greifensee, ensemble learning, fresh water, lake plankton images

## Abstract

Plankton are effective indicators of environmental change and ecosystem health in freshwater habitats, but collection of plankton data using manual microscopic methods is extremely labor-intensive and expensive. Automated plankton imaging offers a promising way forward to monitor plankton communities with high frequency and accuracy in real-time. Yet, manual annotation of millions of images proposes a serious challenge to taxonomists. Deep learning classifiers have been successfully applied in various fields and provided encouraging results when used to categorize marine plankton images. Here, we present a set of deep learning models developed for the identification of lake plankton, and study several strategies to obtain optimal performances, which lead to operational prescriptions for users. To this aim, we annotated into 35 classes over 17900 images of zooplankton and large phytoplankton colonies, detected in Lake Greifensee (Switzerland) with the Dual Scripps Plankton Camera. Our best models were based on transfer learning and ensembling, which classified plankton images with 98% accuracy and 93% F1 score. When tested on freely available plankton datasets produced by other automated imaging tools (ZooScan, Imaging FlowCytobot, and ISIIS), our models performed better than previously used models. Our annotated data, code and classification models are freely available online.

## 1. Introduction

Plankton are a key component of the Earth's biosphere. They include all the aquatic organisms that drift along with the currents, from tiny bacteria and microalgae, to larvae of vertebrates and invertebrates. Photosynthetic phytoplankton are responsible for about half of the global primary production (Behrenfeld et al., 2001) and therefore play a central role in atmospheric carbon fixation and oxygen production. Zooplankton are a broad group of aquatic microorganisms, spanning over tens of thousands of species (Sournia et al., 1991), and comprising both carnivores and herbivores, the latter feeding on phytoplankton. Plankton are a critical component of aquatic food-webs, producing organic matter that forms the ultimate source of mass and energy for higher trophic levels (Lotze et al., 2019), and serve as food for fish larvae (Banse, 1995). The death and excretion of planktonic organisms results in massive amounts of carbon being sequestered, regulating the biological carbon pump locally and globally (Volk and Hoffert, 1985). Plankton biodiversity and dynamics therefore directly influence climate, fisheries and the sustenance of human populations near water bodies.

Planktonic organisms, being mostly small in size, have short lifespans and a strong sensitivity to environmental conditions, which makes their diversity and abundances very effective indicators of environmental change and ecosystem health. Particularly in freshwater ecosystems, they suffer from combined exposure to human local impacts and global change, such as warming and invasive species (Williamson et al., 2009). Information on individual plankton species is also critically important for the monitoring of harmful algal blooms, which can cause huge ecological and economical damage and have severe public health consequences (Huisman et al., 2018). The diversity and abundance of plankton is generally measured using labor intensive sampling and microscopy, which suffer from a number of limitations, such as high costs, specialized personnel, low throughput, high sample processing time, subjectivity of classification and low traceability and reproducibility of data. These limitations have stimulated the development of a multitude of alternative and automated plankton monitoring tools (Lombard et al., 2019), some of which were recently applied in freshwater systems (Spanbauer et al., 2020; Merz et al., 2021; Tapics et al., 2021). Recently developed methods like eDNA hold a lot of promise in particular to monitor biodiversity at large spatial and temporal scales, to identify cryptic species (not detectable morphologically), and to account for genetic/functional diversity (Deiner et al., 2017) but are not yet implemented for high frequency on-site monitoring.

If, on one side, studying freshwater environments offers the opportunity to approach several issues related to (i) automated recognition of plankton taxa in systems that are heavily monitored for water quality, and (ii) the creation of plankton population time series useful for both research and lake management, on the other side it presents a series of practical advantages. The number of species present in a lake is in the order of few hundreds and community composition changes at the scale of decades (Pomati et al., 2012), and virtually all lakes of the same region tend to share the same geographic/climatic region and the same species pool of plankton taxa (Monchamp et al., 2019). This would allow us to process real Lake data with a diminished need to account for species variability, build rather quickly a database that comprises all seen taxa, and easily use our models for more than one site. Moreover, lakes are usually characterized by lower levels of non-planktonic suspended solids (e.g., sand, debris) compared to coastal marine environments, so one can expect to work with cleaner images, with a relatively small number of non-biological or non-recognizable objects being detected.

Among automated plankton monitoring approaches, imaging techniques have the highest potential to yield standardized and reproducible quantification of abundance, biomass, diversity and morphology of plankton across scales (Lombard et al., 2019; Merz et al., 2021). Currently, several *in-situ* digital imaging devices exists such as, Imaging FlowCytobot (Olson and Sosik, 2007), Scripps Plankton Camera (SPC) (Orenstein et al., 2020), Video Plankton Recorder (Davis, 1992), SIPPER and a dual-magnification modified SPC (www.aquascope.ch) (Merz et al., 2021).

These digital imaging systems can produce very large volumes of plankton images, especially if deployed *in-situ* for automated continuous monitoring (Orenstein et al., 2020; Merz et al., 2021). While the extraction of image features that describe important plankton traits like size and shape are well-established (Orenstein et al., 2020; Merz et al., 2021), classifying large volumes of objects into different plankton taxonomic categories is still an ongoing challenge, and represents the most important component for plankton monitoring (MacLeod et al., 2010). Automated classification of imaged plankton objects may help taxonomists annotating images and allow sampling and counting taxa at high temporal and spatial resolution. Automation of plankton monitoring could represent a key innovation in the assessment and management of water quality, aquatic biodiversity, invasive species affecting ecosystem services (e.g., parasites, invasive mussels), and early warning for harmful algal blooms.

Automated plankton classification is characterized by a set of features that make this task less straightforward than other similar problems. The data sets used for training, as well as the images analyzed after deployment, cover wide taxonomic ranges that are very unevenly distributed (some taxa are very common and others are rarely seen - this is called *data imbalance* or *class imbalance*) (Orenstein et al., 2015), and this distribution changes over time, e.g., with new taxa appearing or disappearing, or a different life stages of a species dominating the signal (Schröder et al., 2018). Moreover, many images do not belong to any taxon (e.g., dirt), or they cannot be identified due to the low resolution, their position, focus, or being cropped. Furthermore, labeling these data sets requires a high effort, because they need to be annotated by expert taxonomists, and sampling images from videos, as it is done e.g., for camera traps (Tabak et al., 2019), is not helpful because the alignment of the organisms with respect to the camera does not generally change throughout the exposure time.

Image classification models fall into several broad categories, including unsupervised models (which clusters and classifies images without any manually-assigned tags), supervised models (which use a training library of manually identified images to develop the classification model), and hybrid models (which combine aspects of supervised and unsupervised learning). Even though there is current research that relies on unsupervised learning (Salvesen et al., 2020; Schröder et al., 2020) or on the development of specific kinds of data preprocessing (Zhao et al., 2010; Zheng et al., 2017), the current state of the art for classifying plankton data sets most often involves deep convolutional neural networks trained on manually classified images (Dai et al., 2016; Dai et al., 2017; Lee et al., 2016; Li and Cui, 2016; Py et al., 2016; Orenstein and Beijbom, 2017; Cui et al., 2018; Dunker et al., 2018; Luo et al., 2018; Rodrigues. et al., 2018; Bochinski et al., 2019; Lumini and Nanni, 2019; Eerola et al., 2020; Kerr et al., 2020; Lumini et al., 2020; Guo et al., 2021; Henrichs et al., 2021)^1^, which allow for a great flexibility across applications and were demonstrated more satisfactory than relying on the manual extraction of features (González et al., 2019). These applications very often resort to transfer learning (Tan et al., 2018), which consists of using models which were pretrained on a large image dataset [usually, ImageNet (Deng et al., 2009)], and adapting them to the specific image recognition problem. Transfer learning requires comparatively less human annotated data in the target domain to get a reasonable model after training than the model trained from the scratch. It also speeds up the training process and results in a better performing model. Transfer learning was used in a two-step process to deal with data imbalance (Lee et al., 2016), but most commonly it is used because it allows for the training of very large models in reasonable times. The main differences in the various applications to plankton often dwell in the kind of image preprocessing. For example, Dai et al. (2017) filters the images in different ways, and feeds both the original and the filtered images as input to the models, Cui et al. (2018) applies logarithmic image enhancement on black and white images, and Lumini and Nanni (2019) tests different ways of resizing the pictures.

Furthermore, several models can be used in synergy in order to obtain better performances (be it to deal with data imbalance or to reach a higher weighted accuracy). Two main approaches to combining multiple models are collaborative models and ensembling. The former consists of training models together to produce a common output (Dai et al., 2017; Kerr et al., 2020), while the latter trains the models separately and combines the outputs in a later stage. Collaborative models were used recently to counter data imbalance, yielding high performances on single-channel (i.e., black and white) images obtained in Station L4 in the Western English Channel (Kerr et al., 2020). However, this involves deploying simultaneously several models, resulting in a very high memory usage, unless one uses smaller versions of the typically used models (thus, not allowing for transfer learning). Ensembling allows to fuse virtually any number of learners, and resulted in very satisfactory performances when joining different architectures (where DenseNets most often do best) or kinds of preprocessing (Lumini and Nanni, 2019).

The mentioned methods for automated plankton classification were principally deployed in salt-water coastal habitats. To our knowledge, the only previous work performing image classification on freshwater images is Hong et al. (2020), where the data does not come from an automated system, and they study a small balanced dataset sorted in four categories (daphnia, calanoid, female cyclopoid, male cyclopoid), and obtain a maximum classification accuracy of 93%.

In this paper, we study the classification of plankton organisms from lake ecosystems, on a novel dataset of lake plankton images that we make freely accessible, together with a code that allows to easily train and deploy our deep neural networks. We analyze plankton images from the Dual-magnification Scripps Plankton Camera (DSPC), which is a dark field imaging microscope, currently deployed in Lake Greifensee (Switzerland) (Merz et al., 2021), and specifically the images from the 0.5x magnification, which targets zooplankton and large colony-forming phytoplankton taxa in the ranges of 100 μm to 1 cm. We manually annotated 17943 images consisting of *n*_*c*_ = 35 unevenly distributed categories (classes), which were collected *in-situ* using the DSPC deployed at 3 m depth in Lake Greifensee. We propose a set of deep learning models that makes use of transfer learning, and we combine them through versions of collaborative and ensemble learning. In particular we explore several ways to ensemble our models based on recent findings in statistics (D'Ascoli et al., 2020; Geiger et al., 2020). We evaluate the performances of our models on publicly available datasets, obtaining a slight but systematic increase in performance with respect to the previous literature. The simplest of the presented models were used to analyze part of the data in Merz et al. (2021).

## 2. Materials and Methods

### 2.1. Data Acquisition

**Figure 1:**
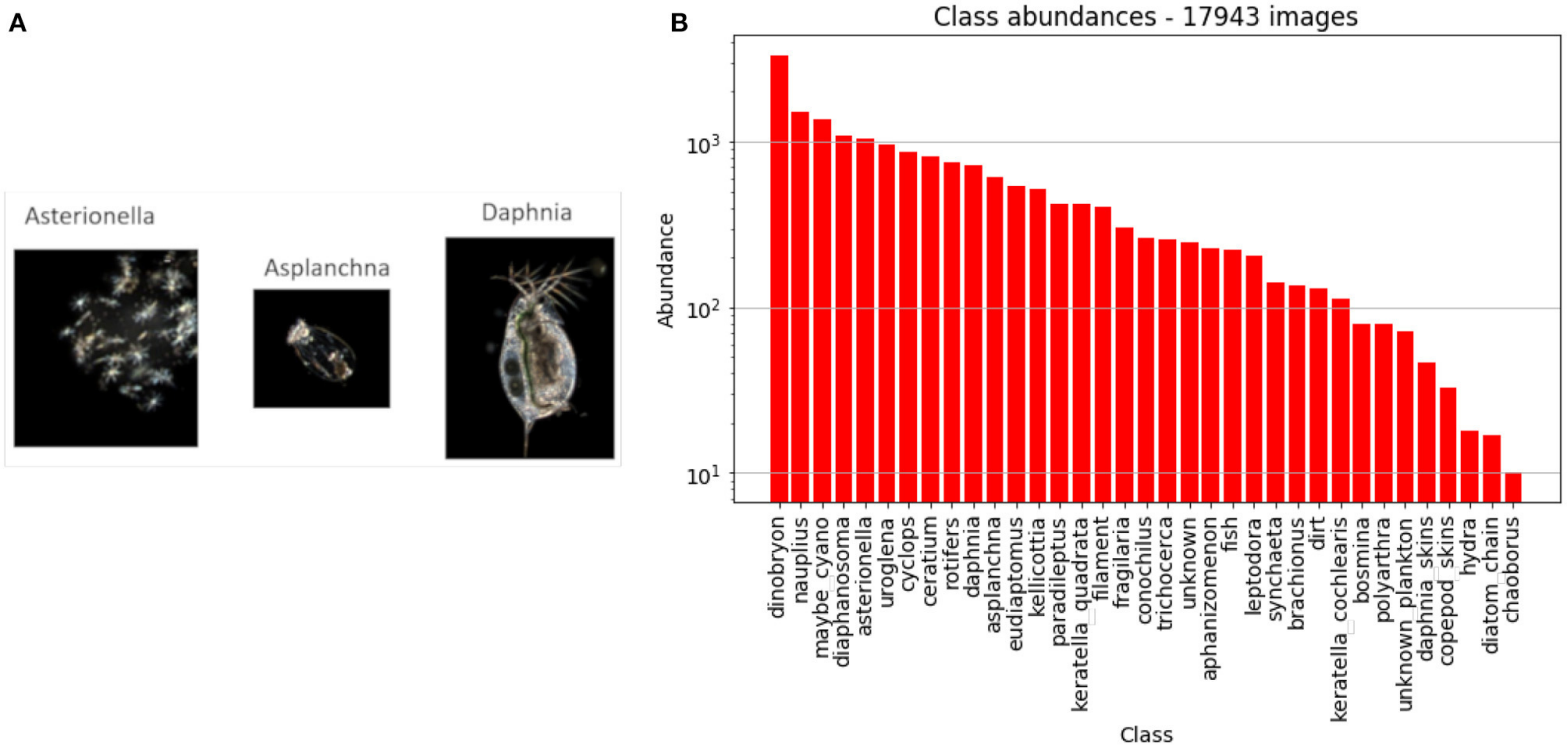
**(A)** Sample images from the DSPC in Lake Greifensee. **(B)** Abundance of each class in our dataset. The word *class* is intended in the classification sense, and does not indicate the taxonomic rank. Note that the *y* axis has a logarithmic scale.

We used images coming from the DSPC (Merz et al., 2021), deployed in Lake Greifensee, and acquired from wild plankton taxa across the years 2018 to 2020^2^. The DSPC takes images of the microscopic plankton taxa at user-defined frequencies and time intervals (for more details and camera settings see Merz et al., 2021). The original full frame images may contain from zero to several images of planktonic organisms, as well as non-organic matter. The full frames are segmented on site in real time, and regions of interest (ROIs), which contain e.g., plankton organisms, are saved and used for image feature extraction and classification. Images of objects at the boundary of the vision range of the camera result cropped, but we keep them anyway, as most of the time we are still able to identify them. The images have a black background, which favors the detection of ROIs. These have different sizes depending on the size of the detected object. For each ROI, we extracted 64 morphological and color features, and performed a series of graphical operations to make the image clearer^3^. In Figure 1A we show some examples of what the final images look like. In the Supplementary Section 1, we provide an extensive description of the dataset and all its classes, together with one sample image from each class in Supplementary Figure 1. In the Supplementary Section 2, we describe the afore-mentioned 64 morphological features.

### 2.2. Data Preparation

The DSPC can be run with two different magnifications (Merz et al., 2021), but in this paper we report only on the images taken at the lower magnification, which contain mostly zooplankton taxa and several large colonial phytoplankton. We manually annotated a dataset of 17,943 images of single objects, into *n*_c_ = 35 classes^4^. In Figure 1B we show the names of all the *n*_*c*_ classes, along with the number of labeled images of each class. Note that there are 300 times more annotated images of the most common class (dinobryon) than the rarest class (chaoborus).

### 2.3. Open-Access Availability of Our Dataset

We call ZooLake the described dataset of labeled plankton images. We give extensive details on ZooLake in the Supplementary Sections 1, 2, and made the data openly available online at the following link: https://data.eawag.ch/dataset/deep-learning-classification-of-zooplankton-from-lakes.

### 2.4. Further Data Preparation

Since for most deep learning models it is not convenient to have images of different sizes, we resized our images in such a way that they all had the same size. The two simplest ways of doing this are either by (i) Resizing all the images to 128 × 128 pixels irrespective of its initial dimensions thus not maintaining the original proportions, or (ii) Shrinking them in such a way that the largest dimension is at most 128 pixels (no shrinking is done if the image is already smaller) and padding them with a black background in order to make them 128 × 128. The former method has the disadvantage of not maintaining proportions. The latter has the problem that in images with a very large aspect ratio there is a loss of information along the smallest dimension^5^. The two methods are compared in Lumini and Nanni (2019), where it is seen that procedure (i) gives slightly better performances in most datasets. Further, the information lost when reshaping of the objects' aspect ratios can be recovered by using the initial aspect ratio (and similar quantities) as an extra input feature. For these reasons, the results we show in the main text are all obtained through method (i).

In order to artificially increase the number of training images, we used data augmentation technique of applying random deformations to the training images (Abadi et al., 2016). The transformations we applied, which did not change the data distribution, include rotations up to 180°, flipping, zooming up to 20%, and shearing up to 10%. As for the morphological and color features, we calculated 44 additional ones on unaugmented images (see Supplementary Section 3), and standardized the resulting 111 features to have zero mean and unit standard deviation.

### 2.5. Training, Validation, and Test

We split our images into training, validation and test sets, with a ratio of 70:15:15. All the splits had a distribution of classes similar to the overall data distribution. The exact same splittings were used for all the models. The validation set was used to select the best model (hyper)parameters, while the test set was set aside throughout the whole process, and used only at the very end to assess and compare the performance of all the proposed models.

#### 2.5.1. Performance Metrics

In order to assess the performance of our models, we used accuracy, precision, recall and F1-score. Depending on the specific application, one can be interested in one metric or the other. In this section, we define and briefly explain each one of them, in terms of true positive counts (TP), false positives (FP), and false negatives (FN).

Accuracy. The accuracy, *A*, indicates the number of correct guesses out of the total number of images,


(1) $$\begin{array}{r} A=\frac{\text{total \# of TP}}{\text{total \# of images}}. \end{array}$$

We calculated the accuracy on the whole dataset, without distinguishing classes. This means that the accuracy is dominated by the most present classes.

Precision. When we have a batch of images that have been assigned to a class *i* by our models, we can be interested in knowing how many of those we expect to actually belong to *i*. For this, we use the precision, *P*, that is defined as


(2) $$\begin{array}{r} P=\frac{TP}{TP+FP} \end{array}$$

We first measure the precision related to each single class, and then average the per-class precision. This is called a *macro* average, and it gives every category the same weight. This ensures that this metric is not dominated by the most abundant classes.

Recall. The recall, *R*, related to a class *i* is the fraction of images belonging to class *i* that were correctly labeled,


(3) $$\begin{array}{r} R=\frac{TP}{TP+FN} \end{array}$$

Also in the case of the recall we use macro averages.

F1-Score. The F1-Score combines the messages of precision and recall into a single number, which is the harmonic average between the two:


(4) $$\begin{array}{r} \text{F1 Score}=2\frac{PR}{R+P} \end{array}$$

In order to have a high F1-score for a specific class, the predictions of classifier need to have both high precision and recall (i.e., a low number of FP and of FN). Also for the F1-score we report macro averages.

### 2.6. Deep Learning Architectures

A common challenge when choosing deep learning architectures is how to best jointly scale architecture depth, width and image resolution. A recent solution was given in Tan and Le (2019), that proposes a scaling form for these three variables simultaneously, together with a baseline model, called EfficientNetB0, for which this scaling is particularly efficient. This results in better performances than previous state of the art models, with a smaller investment in terms of model parameters and number of operations. The provided scaling form allows us to obtain efficiently scaled models according to how many computational resources we are willing to invest. These models, ordered by increasing size, are called EfficientNetB1, EfficientNetB2, EfficientNetB3, EfficientNetB4, EfficientNetB5, EfficientNetB6, and EfficientNetB7. Given the aforementioned large efforts to apply deep learning models to plankton classification, we believe that it is worth to assess the performances of these architectures on plankton recognition. Aside from those, we also test other deep neural network architectures, some of which were already used successfully for our kind of problems.

**Figure 2:**
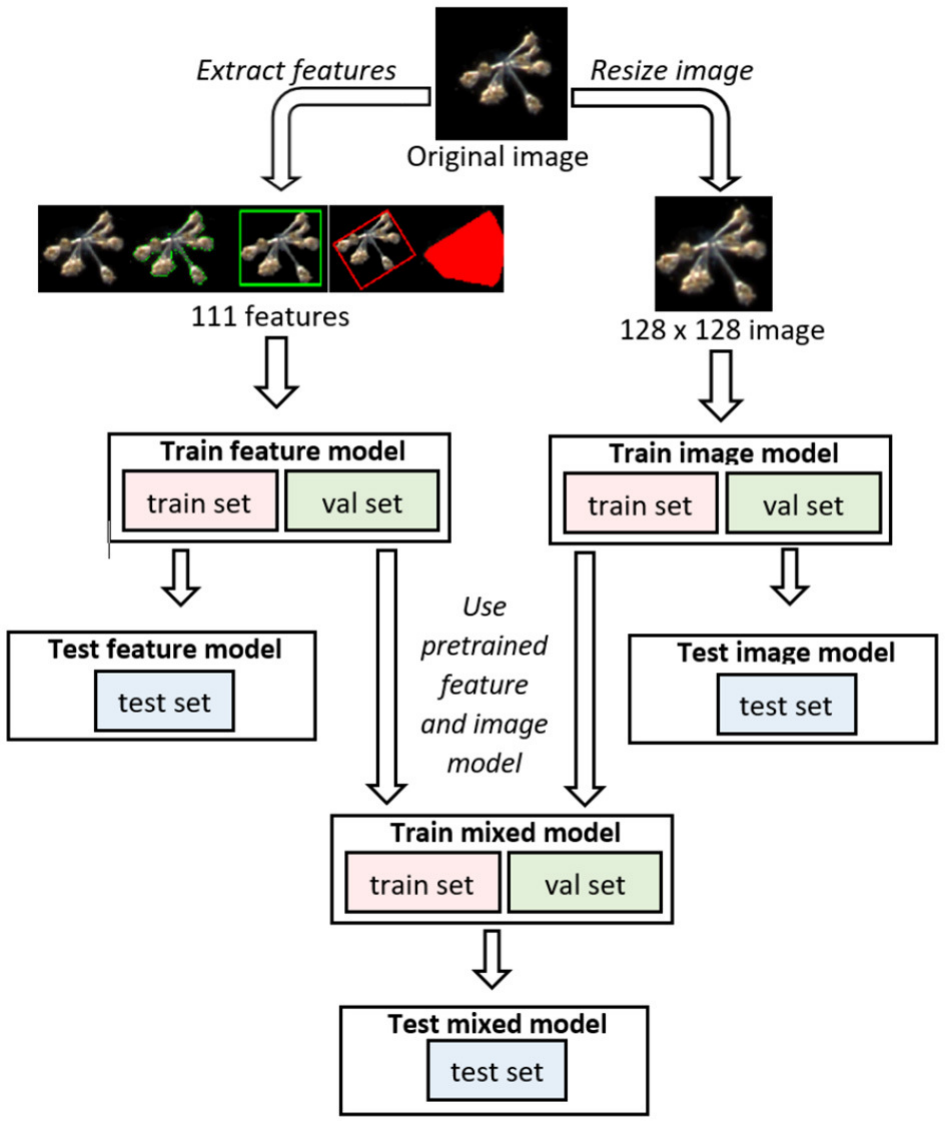
Diagram of the three main kinds of models that we mention in our paper. Image models are convolutional networks that receive only images as input, feature models are multi-layer perceptrons (MLPs) that receive as input only features extracted from the image, but not the image *per se*, and Mixed models join and fine-tune Image and Feature models.

In the main text of this manuscript, we report on 12 different models. These are the EfficientNets B0 through B7 (Tan and Le, 2019), InceptionV3 (Szegedy et al., 2015), DenseNet121 (Huang et al., 2016), MobileNet (Sandler et al., 2018) and ResNet50 (He et al., 2015), trained with transfer learning (section 2.7). Each individual model was trained four times, with different initial conditions from the same parameter distribution^6^. Additionally, we trained multi-layer perceptrons (MLPs) using as input the 111 morphological and color features mentioned in section 2.4, and trained Mixed (collaborative) models that combine the MLPs with a larger model trained on images (section 3.2). In Figure 2 we sketch the structure of these Mixed models. Finally, we also trained 4-layer convolutional networks, to assess whether through specific kinds of ensembling we could reach performances that match larger models (Supplementary Section 5).

### 2.7. Transfer Learning

Since training the mentioned models is a very demanding computational task, we used transfer learning, which consists of taking models that were already trained for image recognition on ImageNet, a very large dataset of non-planktonic images (Russakovsky et al., 2015)^7^. We loaded the pretrained model and froze all the layers. We then removed the final layer, and replaced it with a dense layer with *n*_*c*_ outputs, preceded and followed by dropout. The new layers (dropout, dense, dropout, softmax with categorical cross-entropy loss) and learning rate were optimized with the help of the keras-tuner (O'Malley et al., 2019). We ran the keras-tuner with Bayesian optimization search^8^, 10 trials and 100 epochs, to find the best set of hyperparameters from the Bayesian search. Then, we trained for 200 epochs and used early stopping, i.e., interrupting the training if the validation loss did not improve for 50 epochs, and keeping the model parameters with the lowest validation loss. We then fine-tuned the model by unfreezing all the parameters and retraining again with a very low learning rate, η = 10^−7^, for 400 epochs.

### 2.8. Ensemble Learning

Ensemble methods use multiple independent learning algorithms to obtain better predictive performance than could be obtained from any of the constituent learning algorithms alone, often yielding higher overall classification metrics and model robustness (Seni and Elder, 2010; Zhang and Ma, 2012). For our study we made use of two ensembling methods: averaging and stacking.

#### 2.8.1. Averaging

For every image, the output of a single model is an *n*_*c*_-dimensional confidence vector representing the probability that the model assigns to each class. The model's prediction is the class with the highest confidence. When doing average ensembling over *n* models, we take the average over the *n* confidence vectors, and only afterwards choose the class with the highest confidence. With this procedure, all the models contribute equally to the final prediction, irrespective of their performance. We performed average ensembling on the following choices of the models:

1. Across different models, as for example it was successfully done for plankton recognition in Lumini and Nanni (2019) and Lumini et al. (2020).
2. Across different instances of the same model, trained independently 4 times. This is inspired by the recent observation that this kind of averaging can lead to a better generalization in models with sufficiently many (but not too many) parameters (D'Ascoli et al., 2020). We provide a deeper discussion in the Supplementary Section 5.
3. Manual selection of the six best individual models (on the validation set) over all the models. These best models resulted to be DenseNet121, EfficientNetB2, EfficientNetB5, EfficientNetB6, EfficientNetB7 and MobileNet. For each, we chose the initialization that gave the best validation performance. We call this the *Best_6_avg* ensemble model.

#### 2.8.2. Stacking

Stacking is similar to averaging, but each model has a different weight. The weights are decided by creating a meta-dataset consisting of the confidence vectors of each model, and training a multinomial logistic regression on this metadataset. We performed stacking both across initial conditions and across different architectures. We call *Best_6_stack* the ensemble model obtained by stacking the six individual best models (these are the same models that we used for the *Best_6_avg* model).

## 3. Results

### 3.1. Performances

In Table 1, we summarize the performance of the individual models, along with the various forms of ensembling described in section 2.8.

**Table 1:** Test accuracy and F1-score of the individual models across four different initial conditions.

**Model type**	**Model name**	**Initial condition 1 (Accuracy/F1-score)**	**Initial condition 2 (Accuracy/F1-score)**	**Initial condition 3 (Accuracy/F1-score)**	**Initial condition 4 (Accuracy/F1-score)**	**Average ensemble (Accuracy/F1-score)**	**Stacking ensemble (Accuracy/F1-score)**
Feature	MLP	0.910/0.747	0.912/0.768	0.910/0.748	0.909/0.723	0.915/0.762	0.909/0.752
Image	EfficientNetB0	0.956/0.858	0.963/0.884	0.964/0.892	0.964/0.869	0.971/0.905	0.968/0.907
	EfficientNetB1	0.956/0.848	0.958/0.866	0.966/0.893	0.963/0.892	0.970/0.902	0.968/0.897
	EfficientNetB2	*0.967/0.893*	0.967/0.899	0.968/0.894	0.966/0.889	0.975/0.915	0.969/0.913
	EfficientNetB3	0.958/0.841	0.957/0.880	0.959/0.877	0.958/0.868	0.969/0.904	0.965/0.883
	EfficientNetB4	0.958/0.876	0.964/0.870	0.962/0.874	0.962/0.873	0.972/0.903	0.970/0.907
	EfficientNetB5	*0.965/0.879*	0.967/0.892	0.963/0.854	0.959/0.850	0.971/0.891	0.970/0.899
	EfficientNetB6	0.964/0.880	*0.965/0.879*	0.968/0.897	0.964/0.865	0.971/0.904	0.970/0.912
	EfficientNetB7	0.966/0.885	0.970/0.899	0.967/0.886	*0.969/**0.900***	0.974/0.913	0.971/0.909
	InceptionV3	0.965/0.876	0.961/0.883	0.954/0.867	0.964/0.884	0.972/0.901	0.971/0.913
	DenseNet121	0.958/0.859	0.962/0.821	**0.971**/0.861	*0.968/0.890*	**0.976/0.916**	0.975/0.884
	MobileNet	0.960/0.875	*0.959/0.891*	0.958/0.886	0.965/0.870	0.971/0.907	0.971/0.907
	ResNet50	0.962/0.878	0.955/0.853	0.959/0.858	0.959/0.837	0.974/0.908	0.970/0.889
Image ensemble	Average	0.976/0.911	0.977/0.923	0.975/0.909	0.976/0.914	0.977/0.919	
	Stack	0.975/0.908	0.976/0.919	0.976/0.914	0.977/0.915		0.978/0.921
	*Best_6_avg*	0.978/0.924					
	*Best_6_stack*	**0.979/0.927**					

*The rightmost and the bottom lines describe the performance of our ensemble models. The stacked model over all 48 image models performs stacking only once (we do not do two rounds of stacking). The entries in italics represent the six models that we chose for Best_6_avg and Best_6_stack based on the validation F1-score (therefore their performance on the test set is not necessarily the best). In bold, we represent the overall best for each sector*.

We categorize the models in three ways, according to the kind of data they take as input. *Feature models* take numerical features extracted from the images, *image models* take the processed image, and *mixed models* take both features and image.

#### 3.1.1. Individual Model Performance

First, we focus on the performances of the single models. Already the MLP, our simplest model, which does not take the images as input, had a best accuracy of 91.2%. However, the F1-score below 80% reveals that the accuracy is driven by the predominant classes.

All the image models performed better than the MLP both in terms of accuracy and F1-Score. The model with the best F1-score is the EfficientNetB7 (F1 = 90.0%), followed by the EfficientNetB2, which obtained almost the same value, but with a much smaller number of parameters (8.4 × 10^6^ parameters instead of 6.6 × 10^7^ parameters for EfficientNetB7)^9^. The lightest of the models we present is the MobileNet, with around 3.5 × 10^6^ parameters, with a maximum F1-score of 89.1%.

We tried to further improve the performance of EfficientNets by adopting basic methods for dealing with class imbalance. We reweighted the categories according to the number of examples of each class, in order to give an equal weight to all of them despite the class imbalance. We did not notice sizable improvements, so we restricted to only two models. We report on this in the Supplementary Section 4.

#### 3.1.2. Ensembling Across Initial Conditions

As we discuss in the Supplementary Section 5, ensembling across initial conditions can help reduce the generalization gap (i.e., the difference between train and test performance). This was shown for average ensembling (D'Ascoli et al., 2020; Geiger et al., 2020), but we also tested it for stacking. We see that (rightmost columns of Table 1), both for stacking and averaging, this kind of ensembling improves the overall result compared to each individual model's performance. We also show this in Supplementary Figures 4C,D, where in each column we show the performances of all the repetitions of a single model, as well as the result of ensembling through initial conditions. Average ensembling over (only four) initial conditions is very successful for some specific models such as EfficientNetB2 and DenseNet121.

#### 3.1.3. Ensembling Across Models

We also ensembled across available models. For consistency, we first used only one initial condition per architecture (randomly picked, without repetitions). The results shown in Table 1 and Supplementary Figures 4A,B (first four columns of each plot) display a clear improvement when performing this kind of ensembling, which in most cases seems more effective than over initial conditions.

#### 3.1.4. Overall Ensembling

Finally, we ensembled over all models and initial conditions, obtaining a further small improvement. We obtained a slightly better improvement when ensembling on the six best models of the validation set (*Best_6_avg* and *Best_6_stack*), which had the further advantage of requiring less resources than using all 48 models. Our final best image model, *Best_6_stack*, has an accuracy of 97.9%, and an F1-score of 92.7%.

**Figure 3:**
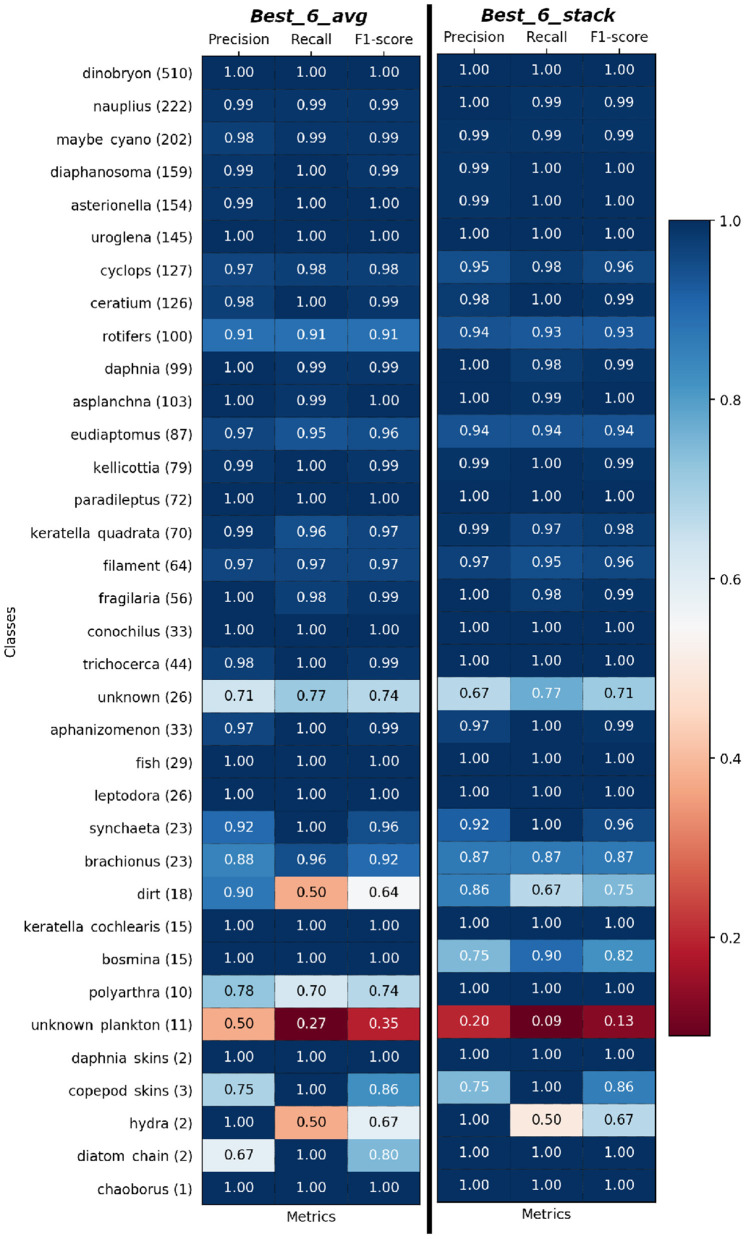
Per-Class precision, recall, and F1-score of *Best_6_avg* **(left)** and *Best_6_stack* **(right)** model on test set sorted based on Figure 1B. Support i.e., the number of samples of the true response that lies in each class of target values is given beside class name.

Toward practical purposes, the performances of *Best_6_avg* and *Best_6_stack* are even better than they appear if we take into account the nature of our dataset: the dataset is imbalanced, and for the most numerous two thirds of the classes we have almost perfect classification, as shown in Figure 3, where we show the per-class performances. For the remaining third, the minority classes, the performance is good, though less reliable due to the very low number of test images at hand. If we keep into account the number of available images, the only three classes with a lower performance are the container (or junk) classes: unknown, dirt, unknown_plankton^10^. This is not surprising, since these classes contain a wide variety of different objects, and it is less of a problem from the point of view of plankton monitoring, since misclassifications involving these classes are less relevant (we show the confusion matrices in the Supplementary Figure 5).

If we exclude the three junk classes (unknown, dirt, unknown_plankton), we reach F1-score=97.3%. If we only consider the 23 classes for which the ZooLake dataset contains at least 200 examples (and keep the junk classes with ≥200 examples), the F1 scores go up to 98.0%. Finally, if we both exclude the classes with less than 200 examples and the junk classes, we obtain F1-score=98.9%.

Moreover, even when making mistakes, our models are not completely off. We can see this in Table 2, where we plot the top-2 metrics of the *Best_6_avg* and *Best_6_stack* models. These represent how good the models' guesses are if the second choice of the classifier is considered as a success. We see that the macro-averaged recall increases by 3%, and the total number of misclassified images is halved, with the top-2 accuracy exceeding 99%.

**Table 2:** Top-1 and top-2 recall and accuracy.

**Model**	**Macro recall**	**Accuracy**
*Best_6_avg* (top-1)	0.926	0.978
*Best_6_avg* (top-2)	0.958	0.992
*Best_6_stack* (top-1)	0.928	0.979
*Best_6_stack* (top-2)	0.947	0.988

*Top-n scores treat true positives and false negatives based on the n highest values of the confidence vector. In other words, the top-2 scores are the model performances in the case that either of the top two guesses is correct*.

### 3.2. Mixed Models

Since our image preprocessing did not conserve information on the image sizes, we trained mixed models that took as input a combination of image and 111 numerical features calculated from the image.

The numerical features were fed into the MLP described in the Supplementary Section 3, while the images were given as input to one of the image models described in Table 1. The two models were then combined and fed into a dense layer, followed by a softmax with categorical cross-entropy loss.

With both features and images (and no image augmentation) as input we trained with a low learning rate η = 10^−5^ for 400 epochs. For each choice of the initial conditions, each single image model was combined with its corresponding feature model (an MLP). In total, we trained 12 mixed models for 4 initial conditions each, so 48 mixed models in total.

Then, we ensembled through models and initial conditions in the same way as with the image models described in section 2.8. The test performance of the mixed models is shown in Table 3. The single-model performances are slightly better than those obtained through image-only models (Table 1). However, after ensembling, the performance of mixed models becomes quite similar to that of image models. The best F1 score of the mixed models improves that of the image models by 0.3%, reaching 93.0%.

**Table 3:** Mixed model test accuracy and F1-score of the individual models across four different initial conditions.

**Model type**	**Model name**	**Initial condition 1 (Accuracy/F1-score)**	**Initial condition 2 (Accuracy/F1-score)**	**Initial condition 3 (Accuracy/F1-score)**	**Initial condition 4 (Accuracy/F1-score)**	**Average ensemble (Accuracy/F1-score)**	**Stacking ensemble (Accuracy/F1-score)**
Mixed	EfficientNetB0+MLP	0.962/0.874	0.969/0.857	0.968/0.867	0.966/0.882	0.973/0.917	0.963/0.856
	EfficientNetB1+MLP	0.965/0.872	0.967/0.890	0.970/0.899	0.968/0.860	0.972/0.908	0.964/0.856
	EfficientNetB2+MLP	0.971/0.906	0.969/0.899	0.971/0.907	0.970/0.906	**0.976/0.917**	0.965/0.866
	EfficientNetB3+MLP	0.964/0.864	0.965/0.904	0.965/0.897	0.965/0.884	0.971/0.913	0.958/0.829
	EfficientNetB4+MLP	0.967/0.897	0.968/0.864	0.967/0.884	0.968/0.886	0.973/0.909	0.962/0.847
	EfficientNetB5+MLP	0.967/0.894	0.971/0.868	0.968/0.864	0.967/0.878	0.972/0.889	0.964/0.856
	EfficientNetB6+MLP	0.971/0.881	0.971/0.891	0.971/0.897	0.967/0.873	0.974/0.914	0.966/0.863
	EfficientNetB7+MLP	0.969/0.901	**0.973/0.916**	0.973/0.909	0.970/0.896	0.975/0.916	0.964/0.838
	InceptionV3+MLP	0.968/0.878	0.965/0.893	0.962/0.888	0.970/0.896	0.973/0.911	0.965/0.842
	DenseNet121+MLP	0.966/0.878	0.965/0.833	0.972/0.870	0.972/0.881	0.974/0.881	0.962/0.836
	Mobile+MLP	0.964/0.886	0.966/0.899	0.962/0.893	0.970/0.879	0.971/0.904	0.964/0.857
	ResNet50+MLP	0.965/0.861	0.964/0.890	0.963/0.857	0.965/0.856	0.971/0.875	0.964/0.856
Mixed ensemble	Average	0.975/0.917	0.976/0.923	0.976/0.916	0.975/0.912		
	Stack	0.974/0.914	0.976/0.919	0.975/0.912	0.975/0.912		
	Best_6_avg	0.976/**0.930**					
	Best_6_stack	**0.977**/0.925					

*The trained image models and its corresponding feature model in each of the initial conditions were chosen from Table 1. The bottom four lines depict the performances when using the four kinds of ensembling described in the main text. The italics represent the six models that we chose for Best_6_avg and Best_6_stack based on the validation F1-score (therefore their performance on the test set is not always the best). In bold, we represent the overall best for each sector*.

### 3.3. Comparisons With Literature on Public Datasets of Marine Plankton Images

To compare our approach with previous literature, we evaluated our models on the publicly available datasets indicated in Zheng et al. (2017), which reports classification benchmarks on ZooScan (Gorsky et al., 2010) and the subsets of the Kaggle (Cowen et al., 2015) and WHOI (Sosik et al., 2014) plankton datasets. The ZooScan (Gorsky et al., 2010) consists of 3,771 grayscale images acquired using the Zooscan technology from the Bay of Villefranche-sur-mer. It consists of 20 classes with variable number of samples for each class. The Kaggle subset (Zheng et al., 2017) comprises 14,374 grayscale images from 38 classes, acquired by in-situ Ichthyoplankton Imaging System (ISIIS) technology in the Straits of Florida and used for the National Data Science Bowl 2015 competition. The distribution among classes is not uniform, but each class has at least 100 samples. The WHOI subset (Sosik and Olson, 2007) contains 6,600 grayscale images of different sizes, that have been acquired by Imaging FlowCytobot (Olson and Sosik, 2007), from Woods Hole Harbor water samples. The subset contains 22 manually categorized plankton classes with equal number of samples for each class.

We compared the performance of our image models with the best models of Zheng et al. (2017), Lumini and Nanni (2019), and Lumini et al. (2020). For WHOI, we used the exact same train and test sets, since the dataset splitting was available. For ZooScan and Kaggle we used, respectively, two-fold cross-validation and five-fold cross-validation as in Lumini et al. (2020). We used our Best_6_avg and Best_6_stack models, and did transfer learning starting from the weight configurations trained on our ZooLake dataset^11^. We fine-tuned each of the 6 selected models belonging to Best_6_avg and Best_6_stack with a learning rate η = 10^−5^, and followed with average and stack ensembling^12^.

**Figure 4:**
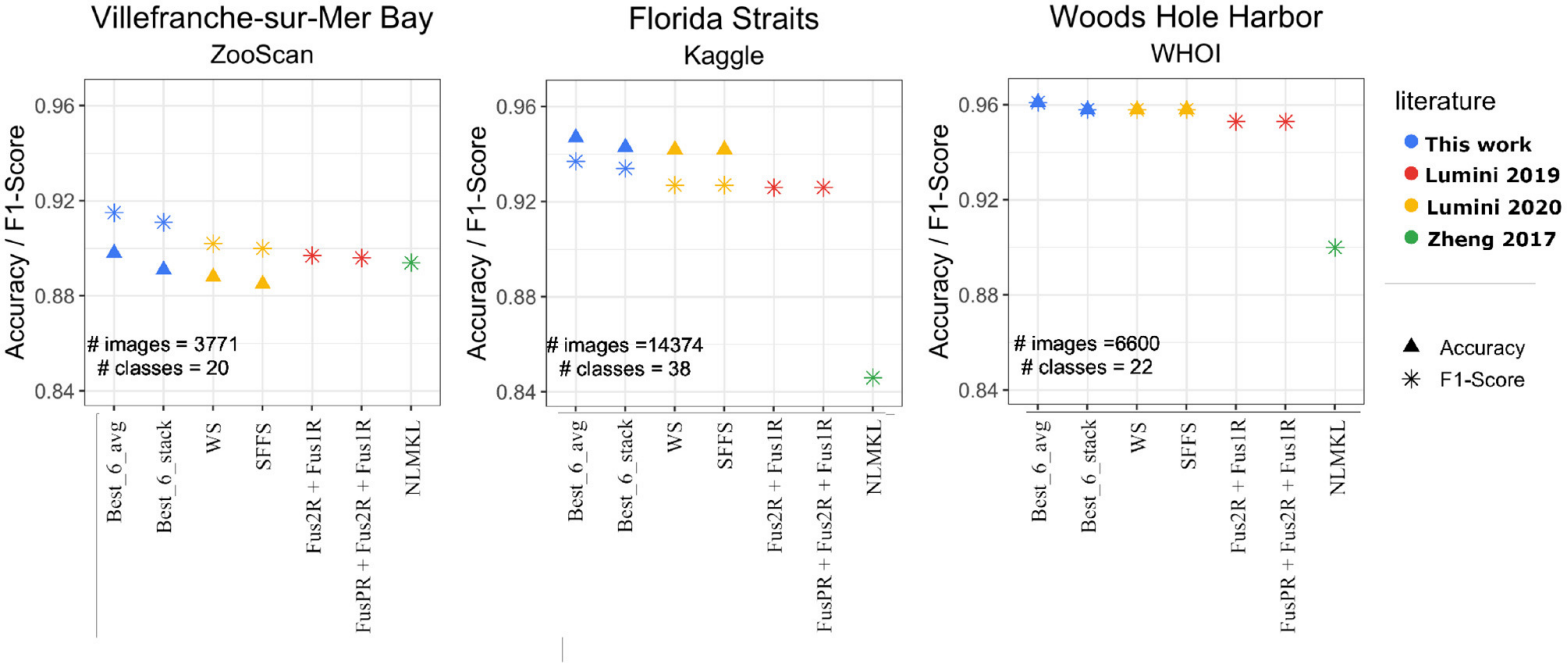
Performances Accuracy/F1-score of our *Best_6_avg* and *Best_6_stack* models (blue points) on the publicly available datasets (ZooScan, Kaggle, WHOI), and comparison with previous results from literature. The yellow points indicate ensemble models from (Lumini et al., 2020): SFFS (Sequential Forward Floating Selection—a feature selection method used to select models), WS (Weighed Selection—a stacking method that maximizes the performance while minimizing the number of classifiers). The red points are the Fus models from (Lumini and Nanni, 2019), which fuse diverse architectures and preprocessing. The green points stand for non-linear multi kernel learning (NLMKL), where an optimal non-linear combination of multiple kernels (Gaussian, Polynomial, and Linear) is learnt to combine multiple extracted plankton features.

As we show in Figure 4, our Best_6_avg and Best_6_stack models performed always slightly better than all the previous methods/studies. The improvement in terms of F1-score is consistent throughout the three datasets, with a 1.3% improvement on the previously best model for ZooScan, a 1.0% on Kaggle, and a 0.3% on WHOI. The same data of Figure 4 is available in the Supplementary Table 2.

Note that these improvements come with a further advantage. Our results require ensembling over a smaller number of models, and of total parameters. The 6-model average ensemble consisted of around 1.58 × 10^8^ parameters compared to the 6.25 × 10^8^ (4.0 times more) of the best model in Lumini et al. (2020) and the 1.36 × 10^9^ (8.6 times more) of the best model in Lumini and Nanni (2019). A major advantage of having lighter-weight models is that it allows for a simpler deployment and sharing with field scientists.

## 4. Discussion

In this paper, we presented the first dataset, to our knowledge, of Lake plankton camera images, and showed that through an appropriate procedure of preprocessing and training of deep neural networks we can develop machine learning models that classify them with high reliability, reaching 97.9% accuracy and 93.0% (macro-averaged) F1-score. These metrics improve to 98.7% accuracy and 96.5% F1-score if we exclude the few container classes (dirt, unknown, unknown_plankton), that do not identify any specific taxon, with the F1 score reaching 98.9% if we further restrict to the two thirds of the categories with a sufficient number of examples^13^.

We trained several deep learning models. Our main novelties with respect to previous applications to plankton are the usage of EfficientNet models, a wise and simple ensemble model selection in the validation step, and the exploration of ensembling methods inspired by recent work in theory of machine learning (D'Ascoli et al., 2020). We checked the utility of using mixed models which as input include, in addition to the image, numerical features such as the size of the detected object, and found that this increases the single-model performance, but the gain is flattened out once we ensemble across several models (though the best F1 score still improved from 92.7 to 93.0%). We also checked whether the performance of the EfficientNets improved by correcting through class imbalance through class reweighting, and found no sizable improvement. We compared the performances of our models with previous literature on salt-water datasets, obtaining an improvement that was steady across all datasets.

The best performing individual models were EfficientNets, MobileNets, and DenseNets. Notably, the performance of the EfficientNets did not scale monotonously with the number of model parameters, perhaps due to the class imbalance of our dataset. The EfficientNets B2 and B7 were the best performing, but B2 uses a smaller number of parameters. If we had to select a single architecture, our choice would lean toward MobileNet or EfficientNetB2, given their favorable tradeoff between performance and model size. If we apply ensembling, averaging and stacking provide similar performances, so we prefer averaging due to its higher simplicity. As for Mixed models, their narrow increase in performance after ensembling does not seem to justify their additional complexity in terms of deployment.

The Scripps Plankton Camera systems are a new technology that allows users to obtain large volumes of high-resolution color images, with virtually any temporal frequency. We noticed that the images that we obtained were clearer than those coming from marine environments (c.f. Orenstein et al., 2020), which favored the process of annotation and classification. Additionally, the taxonomic range is more stable during the seasonal progression compared to marine studies: fewer taxa are present in lake than coastal marine environments, colonization by new taxa are relatively rare at the inter-annual scale (new taxa do not appear often), and lakes of the same region share large part of the plankton community composition. This makes the study of lake plankton dynamics an interesting and more controlled case study for method development due to its relative ecological simplicity and temporal stability, and implies that classifiers for lake taxa are more robust in these environments over space and time. This is particularly important from an application point of view, since the tools we developed in this paper are not only applicable for analyzing plankton population time series in Lake Greifensee, addressing problems such as inferring interactions between taxa and predicting algal blooms, but they may be transferable to other similar lakes. Lakes represent very important water resources for human society and require routine monitoring for water quality and provision of ecosystem services.

The models developed in this study have already been used in real-world monitoring, for part of the counting performed in Merz et al. (2021). We make both the dataset and our code freely available^14^.

## Data Availability Statement

The dataset presented in this study can be found in online repositories. The name of the repository and accession number can be found below: https://doi.org/10.25678/0004DY.

## Author Contributions

MB-J and FP designed the study. SK and MB-J built the models for Zooplankton classification. SK, TH, EM, TB, MR, PI, FP, and MB-J were actively involved in the discussion while building and improving the models. TH, EM, TB, MR, PI, and FP were also involved in annotating the plankton images. All authors contributed to the manuscript.

## Funding

This project was funded by the Eawag DF project Big-Data Workflow (#5221.00492.999.01), the Swiss Federal Office for the Environment (contract Nr Q392-1149), and the Swiss National Science Foundation (project 182124).

## Conflict of Interest

The authors declare that the research was conducted in the absence of any commercial or financial relationships that could be construed as a potential conflict of interest.

## Publisher's Note

All claims expressed in this article are solely those of the authors and do not necessarily represent those of their affiliated organizations, or those of the publisher, the editors and the reviewers. Any product that may be evaluated in this article, or claim that may be made by its manufacturer, is not guaranteed or endorsed by the publisher.

## Footnotes

^1^For a synthetic survey of relatively recent applications of deep and machine learning to plankton classification we refer the reader to Moniruzzaman et al. (2017) and Lumini et al. (2020).

^2^Details on the camera and on the data acquisition can be found in Merz et al. (2021) (and Orenstein et al., 2020 for an analogous camera deployed in the ocean).

^3^For details and code on image preparation we refer the reader to https://github.com/tooploox/SPCConvert. This code contains the pipeline we used, of color conversion, edge-detection and segmentation, morphological feature extraction, foreground masking, and inverse filtering of masked foreground.

^4^Throughout this text, we use the machine-learning connotation of the work “class,” which indicates a category for classification, and not a taxonomic rank. In other words, our classes are not necessarily related to the taxonomic classification of the categories. For example, we call “class” categories like “diatom chain,” “unknown” or also “dirt”.

^5^Imagine that an image is originally 1280 × 50 square pixels. Re-scaling the largest dimension to 128 pixels, maintaining the proportions, implies that there resulting image is only 5 pixels high, which means that we almost completely lose the information contained in the image. Further, with method (ii), the large images are re-scaled, while the small ones are not, so even in this case the image size suffers a non-linear transformation.

^6^All the initial conditions of all models were different realizations from the same distribution. We used a Glorot (or Xavier) uniform initializer, which is a uniform distribution within [−*a, a*], where $a=\sqrt{6/\left(n_{i}+n_{o}\right)}$, and *n*_*i*_ and *n*_*o*_ are, respectively, the number of input and output units in the weight tensor. All the models were trained with the Adam optimizer, a stochastic gradient descent method that is based on adaptive estimation of first-order and second-order moments. We used, respectively, 0.9 and 0.999 as decay rate of the first and second moment estimates.

^7^Transfer learning from models trained on plankton images was tried in Orenstein and Beijbom (2017), but it did not yield better results than using the models trained on ImageNet.

^8^The Bayesian optimization is a trial-and-error based scheme to find the optimal set of hyperparameters (Mockus, 2012).

^9^EfficientNetB7 models also took about 8 h to train, more than twice to train than their lightweight counterpart. We show the times required for hyperparameter tuning and for training in Supplementary Table 3.

^10^We have a fourth container class, maybe_cyano, but in that case we obtain almost perfect classification. See Supplementary Section 1 for a description of this category.

^11^Since our Best_6_avg and Best_6_stack models were originally trained on three-channel image data, we had to adapt WHOI and Kaggle data images as they consisted of single channel images. The single channel was replicated 3 times to have 3 channels image such that they are similar to ZooLake. The ZooScan however had 3 channels images similar to ZooLake.

^12^We stress that for simplicity we used the 6 models that performed best on our ZooLake validation set. Arguably, we could expect an even higher performance if we selected the 6 models on the validation set of each of the three public datasets. We did not do this because it made the reporting more complicated, and our models perform better than the previous literature even in this case.

^13^It is not guaranteed that the performances of a classifier on the test set (i.e., on a hold-out partition of the dataset) are equally good when deployed in the real world (Hand, 2006; Moreno-Torres et al., 2012; Recht et al., 2019). This is because it is hard (or even impossible) to guarantee that a data set consists of completely independent samples which are fully representative of the underlying distribution of images. In particular, this is a source of concern with plankton, where there is a large number of sources of bias, such as environmental conditions, species composition, and annotation bias (González et al., 2017). Therefore, one can expect that our model performances can be lower when deployed for field monitoring. Prescriptions to validate models in a real-life context require large annotation efforts (González et al., 2017, 2019), which we plan to report on in future work.

^14^Our ZooLake dataset can be downloaded at https://data.eawag.ch/dataset/deep-learning-classification-of-zooplankton-from-lakes, and our code is available at https://github.com/mbaityje/plankifier.
