# Supplementary material for: Deep Learning Classification of Lake Zooplankton
Source: Front Microbiol. 2021 Nov 15;12:746297. doi: 10.3389/fmicb.2021.746297 (PMC8634433; doi:10.3389/fmicb.2021.746297)
Supplement: Supplementary file 1 [file Data_Sheet_1.pdf]

Deep Learning Classification of Lake Zooplankton (supplementary material)

1 THE ZOOLAKE DATASET

1.1 Classes

The ZooLake dataset contains 17943 images, sorted in 35 classes. The images were taken with the DSPC camera in lake Greifensee (Switzerland), between year 2018 and 2020. Greifensee is a lake that we have been monitoring for many years and from which we know the plankton communities we can find. During the acquisition of the pictures, we have been sampling weekly for zoo and phytoplankton and identified the samples under a traditional microscope which has helped us learn to identify the pictures.

Most of the classes identify specific plankton taxonomic categories. The low magnification camera used for this classifier was meant to categorize different zooplankton groups, but the high quality images, especially when sharp, has allowed us to be able to identify not only big zooplankton but also rotifers, and colony forming phytoplankton. We aimed at identifying the different categories to the maximum taxonomic resolution possible. In Fig. S1 we show an example of image from each single class. In the following, we describe all the classes in the ZooLake dataset:

aphanizomenon: These filamentous cyanobacteria are rarely solitary, mostly gathered in macroscopic fasciculated colonies, arranged in parallel. The single filaments would be almost impossible to identify to the genus level at the given magnification, but the colony form is very characteristic.

asplanchna: Asplanchna is one of the biggest rotifers in our lake. It is a predator, semi transparent, shaped like a sack and with a well developed corona.

asterionella: A diatom algae that builds stellate colonies. The colony itself is only 90um but it commonly makes aggregates forming the commonly known lake snow which are much bigger and hence seen in the low magnification camera. Interestingly when fixing the sample, following old traditional methods those aggregates are broken and one is not aware of the aggregates.

bosmina: A zooplankton of the order Cladocera. Laterally compressed with almost spherical oblate body form and a characteristic long antennule.

brachionus: Brachionus are rotifers with a sack shape body, and this is dorso-ventrally flattened. The foot is long and not segmented

ceratium: This category refers to the species Ceratium hirundinella. The distribution of the horns, size and color of this dinoflagellate makes it difficult to confuse with other species.

chaoborus: Pictures of the larvae of chaoborus, also commonly known as phantom midge. Chaoborus have the shape of a worm, mostly transparent and a distinguishable head capsule with Antennas.

conochilus: This rotifer forms spherical colonies that are radially oriented, attached in the center.

copepod skins: We distinguished copepod pictures from copepods skins after molting (see also daphnia_skins).

cyclops: Zooplankton from the order of Cyclopoida, with antennas and 2 caudal appendages.

daphnia: Zooplankton, a cladocera commonly known as water flea. Sometimes epiphytes or eggs were also clearly seen on the picture. This genus includes several species.

daphnia skins: We know that Daphnia can molt, leaving the exoskeleton floating on the water several times during his life cycle. To avoid counting the exoskeleton as a Daphnia and hence increasing the real

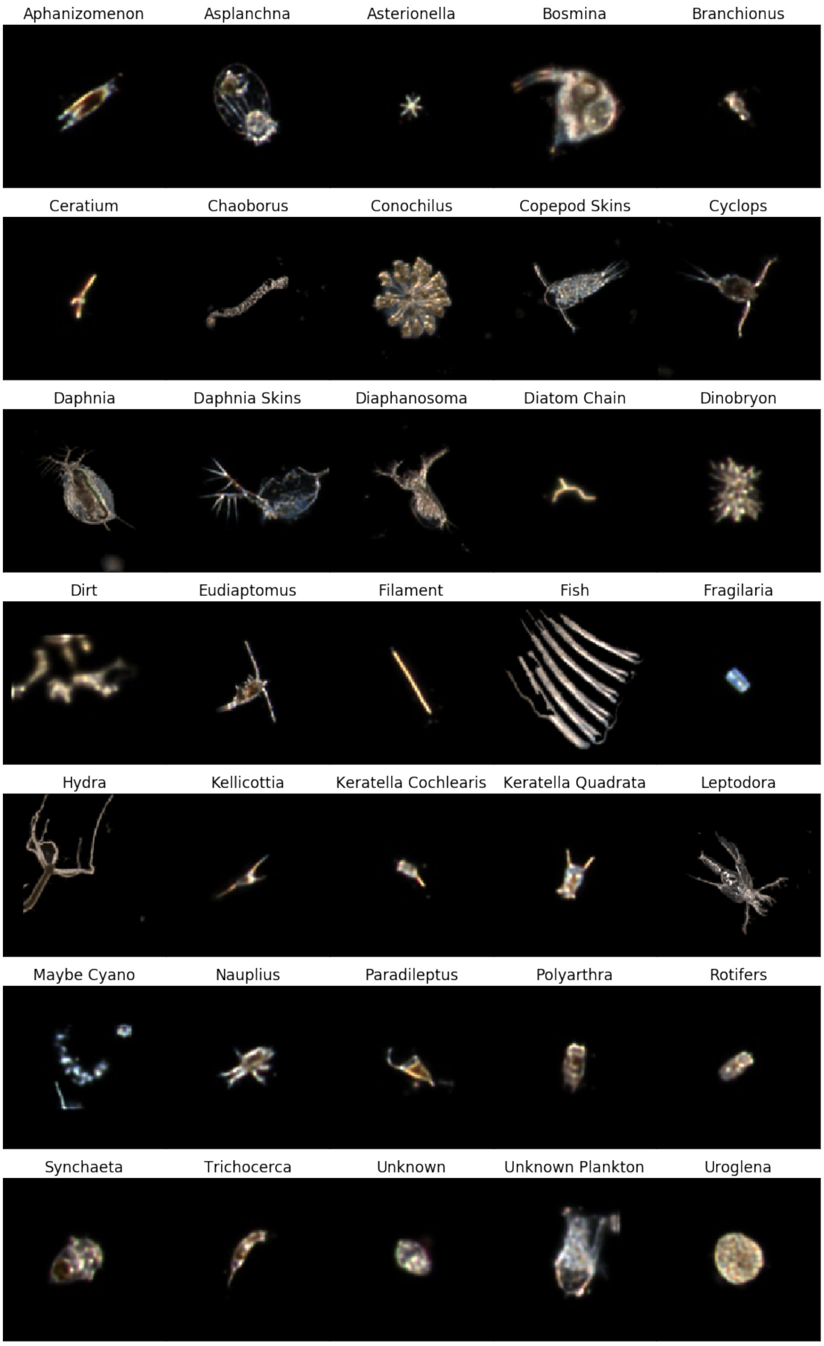

Figure S1. Examples of images from all 35 plankton classes in their original proportion.

Daphnia concentration present in the water we distinguished this class from the daphnia category. The daphnia skins category pictures don't have any organels or eggs inside and are much more transparent.

diaphanosoma: A genus of Cladoceras, zooplankton, similar to daphninds but with a peculiar thicker second Antennas.

diatom chains: In this category we include few genuses of diatoms that make chains like filaments or zig zag. The magnification was not always enough to distinguish those colonies to a higher taxonomic level.

dinobryon: The genus Dinobryon, a Chrysophyceae, is a colony forming algae that includes in this case at least 3 species. Cells in branching colonies, being D. bavaricum a bit more elongated than the other 2 species. Because the colonies are usually dense and big they can be seen in this magnification.

dirt: In this category we included pictures of inorganic and organic material that were clearly not plankton.

eudiaptomus: A zooplankton genus from the order calanoid. The first antennas are very long, more than half of the body. The eggsacks are very distinguishable from the ones from cyclops.

filament: This folder includes different phytoplankton genus of a cylindrical shape and elongated. Appearing as a single filament.

fish: This category includes all pictures were young fishes were partially photographed.

fragilaria: This folder contains the colony forming diatoms *Fragilaria crotonensis* and *Fragilaria capucina*. they both have cells in large ribbon-like colonies. With the low magnification camera it was not possible to differentiate between this 2 species.

hydra: A macroscopic organism of the phylum cnidaria with a single body axis and tentacles. The body is a hollow tube with a gelatinous layer with a texture easy to identify with the camera.

kellicottia: The genus kellicottia is an easy to identify rotifer, with an elongated body and posterior and anterior spines.

keratella cochlearis: This species of rotifer has an oval shape body with a long posterior spine and short anterior spines. It is sometimes difficult to distinguish from the brachionus rotifer, depending on the angle of the picture.

keratella quadrata: In this category the taxonomic resolution of this rotifer is to species level because one can distinguish the 2 caudal spines at the base of the body.

leptodora: A top predator genus of zooplankton that can almost reach the size of 2cm. Most of the pictures of this category were of parts of its antennas or body, but still recognizable.

maybe cyano: This algal category comprises different genuses of Cyanobacteria. All forming gelatinous colonies of different shapes with small cells inside. Due to the light and the darkfield background they look slightly blueish on the DSPC. This class possibly contains also non-organic material, like sand or debris, because they look very similar to some cyanobacteria colonies (especially clathrate microcystis colonies) and are hard to distinguish from one another with this magnification. The gathering of ”maybe cyano” pictures started at blooms of cyanobacteria that were confirmed with the higher magnification camera, where cyanobacteria are more easily recognizable. Then, looking at pictures from the lower magnification camera (the one made for zooplankton and discussed in this paper), the taxonomists could be more sure about tagging the images they thought were cyanobacteria colonies. This is why the folder is called maybe cyano. With the 0p5x magnification it is very difficult to be sure; but with the expert knowledge, and seeing at the other higher magnification pictures taken at the same time, we could confirm that there were many cyanobacteria colonies at that time, which made us learn that cyanobacteria colonies pictures look very similar to the ones on this class, and enabled the tagging.

nauplius: In this zooplankton category we classified the larval stage of all copepods. Nauplii were distinguishable because of their antennas and mandible and an absence of thorax.

paradileptus: The genus paradileptus is a ciliate, has not been seen using traditional fixation methods with Lugol solution, that may had influence on its preservation [1]. It has a conical body with a long tapering, spiraling neck region.

polyarthra: The most important feature of this rotifer is the well developed paddles (or blade-like projections) originating on the body below the head, and the absence of a foot, like in Brachionus

rotifers: In this category we classified smaller rotifers, pictures of other rotifers that did not belong to any of the categories already containing rotifers: brachionus, conochilus, kellicottia, keratella, synchaeta or

trichocerca, or pictures that were either not sharp or not from the right angle to be able to see the features to classify it into another category, since in some cases, in order to identify the rotifer to a genus level, one needs to see a trait that is only seen from a specific plane.

synchaeta: Synchaetas are rotifers with a conical shape body that similarly to trichocerca have foot and toes, but this time those are reduced.

trichocerca: The genus Trichocerca are rotifers with a lorica and a characteristic long 1 or 2 toes emerging from the foot. Anterior spines can be present but are not seen with the current magnification.

unknown: Objects that were for us difficult to decide if they were dirt, or part of zooplankton or algae were ordered into an unknown category.

unknown plankton: In this category we include all objects that we thought could be plankton but that because of the sharpness, focus or angle they were photographed we could not categorize them to a folder with label.

uroglena: A Chrysophyceae algal genus with spherical colonies. They very often occur in blooms, so in very high densities.

1.2 Labeling

The dataset was labeled by a team of 6 taxonomists, listed among the authors of this paper. Every labeled image was checked by at least two taxonomists. As a further check, we adopted a basic active learning procedure, in which the taxonomists occasionally double-checked the labels of the examples that were particularly poorly guessed by our classifiers.

2 FEATURES DESCRIPTION

With the data, we include 64 features of quantities that are directly measured on the raw image. Shape related features and color related features were extracted for every object in the image.[1] The explanations of these features are given below.

2.1 Shape related features:

aspect ratio: The ratio of width to height of the bounding rectangle of the object in the image
eccentricity: This is the ratio of length of minor axis to the length of major axis of an object in the image
major and minor axis length: The longest perpendicular lines that can be drawn through the object in the image
orientation: The overall direction of the object in the image in degrees.
solidity: The ratio of the area of an object to the area of the convex hull of the object. This measures the density of an object.
estimated_volume: This measures the estimated volume of the object in mm^3.
area: This measures the area of the object in real world metric in mm^2.

2.2 Color related features:

We calculated the following properties, for grayscale and colour components (R,G,B):

intensity_mean: The mean average value of the intensity of image pixels for each R, G, B channel
intensity_25_percentile: The first quartile (25%) value of the intensity of image pixels for each R, G, B channel

[1] The code that we used to extract these features from the raw images is available at https://github.com/tooploox/SPCConvert.

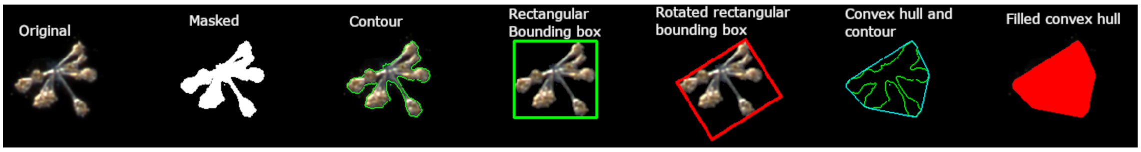

Figure S2. Illustrating some of the features extracted from the object in the image

intensity_50_percentile: The second quartile (50%) value of the intensity of image pixels for each R, G, B channel or in other words median value of the image pixels.
intensity_75_percentile: The third quartile (75%) value of the intensity of image pixels for each R, G, B channel
intensity_std: The standard deviation of the intensity of image pixels for each R, G, B channel - this shows how the pixels vary within the image
intensity_mass_displace: The distance between the peak intensity pixel value to the centre of the mass in an image for each R, G, B channel
intensity_mass_displace_in_images: The mass_displace distance is scaled by the size of the input image for each R, G, B channel
intensity_mass_displace_in_minors: The mass_displace distance is scaled by the minor axis length of the object in the image for each R, G, B channel
intensity_moments_hu: They give information on the shape and the intensity distribution of the image for each R, G, B channel. These Hu moment invariants are invariant to translation, scale and rotation.

3 FEATURE MODELS (MULTI-LAYER PERCEPTRONS)

In addition to the 64 features of the ZooLake dataset, we extracted further features, in order to include some of those proposed in Ref. [2]. This implied adding 44 additional features. These additional features are listed below. The reader can use Fig. S2 to understand some of the definitions.

extent: is the ratio of contour area of the organism to bounding rectangle area.
equivalent Diameter: is the diameter of the circle whose area is same as the contour area of the organism.
image moments: helps to calculate the centroid, area, centre of the mass etc., of the object.
contour_area and contour_perimeter: is the area mm^2 and perimeter mm of the contour of the object.
hull_area hull_perimeter: is the area and perimeter of the convex hull. Convex hull is the smallest convex polygon that can fit in the object.
compactness: is the contour_perimeter squared, divided by $4\pi\times$contour_area. The circle has a compactness of 1.
roundness: is similar to compactness, but here hull_perimeter is used instead of contour_perimeter i.e. $4\pi\times$contour_area divided by hull_perimeter squared. For a perfect circle, the roundness is 1.
w_rot, h_rot and angle_rot: rotated width, height and angle of the rotated bounded rectangle.
rect_width,rect_height and rect_area: width, height and area of the bounded rectangle over the object
rect_area: width $\times$ height.
Convexity: hull_perimeter/contour_perimeter.

Using all 111 features as input, we trained multi-layer perceptron (MLP) models, a basic deep neural network model that alternates layers of matrix multiplication and non-linearities. We ran the Keras tuner [3] to get the optimized number of layers and model hyperparameters by setting Bayesian optimization search, 10 trials and 1000 epochs. As for the other models, this process involved only training and validation sets.

Then, we trained for 200 epochs and with early stopping (with a patience of 50 epochs), and keeping the model parameters with the lowest validation loss. We concluded with a final 400 epochs of training with a learning rate $\eta = 10^{-7}$.

The final feature model consisted of one input layer, one output layer and three hidden layers. The hidden layers consisted of 3 dense had each 0.3 dropout, and had respectively 128, 80 and 80 hidden units, and ReLU, tanh and softplus activations.

4 CORRECTING FOR CLASS IMBALANCE

Given the class imbalance of our dataset, we checked whether a basic method for dealing with data imbalance would improve the performances of our EfficientNet models. We adopted class reweighting, giving minority classes more weight and viceversa [4]. During the training, misclassifications of minority classes are penalized more, and therefore the optimizer will be more keen to correct them. We tested this on two models, EfficientNetB5 and EfficientNetB6, using exactly the same parameters (including those of the Bayesian search) as in *Materials and Methods* section but including class reweighting. As shown in Tab. S1, the F1-score did not improve. Therefore, class reweighting was not explored further.

Model	Accuracy	F1-score
Eff5	0.949	0.838
Eff6	0.958	0.853

Table S1. Class-weightage technique's performance on test data

5 ENSEMBLING OVER INITIAL CONDITIONS

Recent work in Ref. [5] on the double descent peak [6, 7] showed that a major source of the generalization error is initialization variance. This variance can be attenuated by ensembling across different initializations of the same model. This was shown for simple balanced binary datasets in Refs. [5, 8], and was especially useful near the interpolation threshold.[2]

In our case, we did not know where the interpolation threshold was, but we could assume that the models shown in Tab. 1 of the main text are highly over parameterized, and even then models such as Eff4 enjoyed over a 2% improvement thanks to ensembling over only 4 initial conditions.

We can then expect that smaller convolutional networks (thus, closer to the interpolation threshold), can benefit more from ensembling, and we might be able to reach accuracies similar to those of EfficientNets with much simpler models. Therefore, we trained convolutional networks that are close to the interpolation threshold, and investigated the improvement obtained through ensembling. In Fig. S3 we show results from a four-layer convolutional network (conv4) obtained through a Bayesian optimization search, 25 trials, 30 epochs to select the best convolutional network between 2 to 5 convolutional layers, filter size between 32 and 128, kernel size between 8 to 32, dense units between 64 to 256, learning rate between 10^{-2} and 10^{-5}. We used no early stopping here, since ensembling should replace its efficacy [8]. The average final training accuracy that we obtained was 99.24%.

We ran the model with $K = 17$ different initial conditions. Fig. S3 shows the test metrics while ensembling over an increasing number of initial conditions. Blue circles represent the performance of individual conv4 models, whereas the orange dots represent the cumulative performance of average

[2] The interpolation threshold is the ratio between number of parameters and amount of data at which we are able to reach approximately zero error.

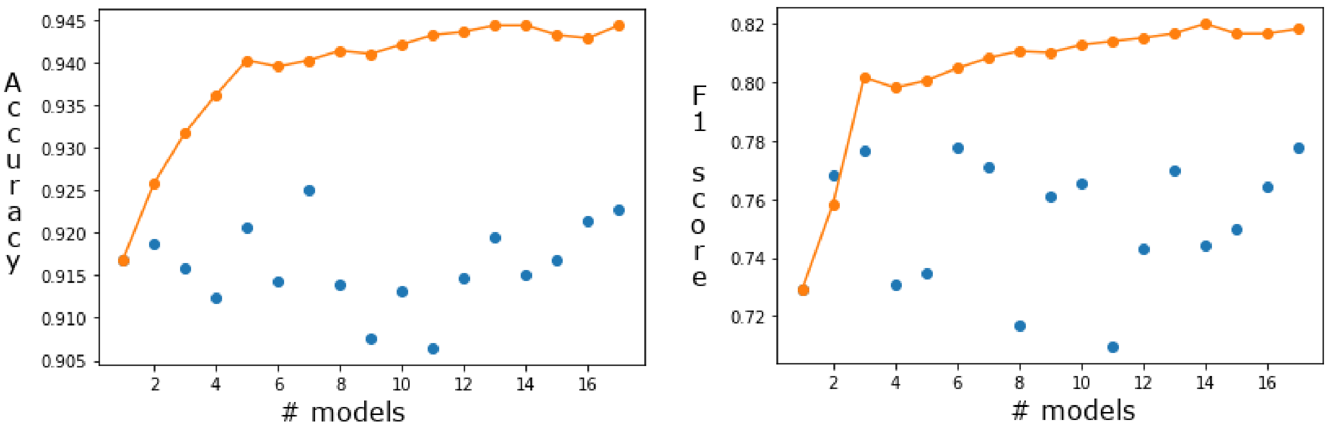

Figure S3. Test accuracy (left) and F1-score (right) of four-layer convolutional networks. Blue dots represent the performance of individual model whereas the orange dots represent the cumulative performance of average ensembles of the model.

ensembles of the model. The benefit of ensembling is clearly visible, as already with five initial conditions we reach an accuracy (F1-score) around 0.94 (0.8). Ensembling over 17 initial conditions give a 0.945 accuracy (0.82 F1-score). This is likely to increase if we increase K, but it does not seem that the performances of the EfficientNets can be reached.

6 SUPPLEMENTARY TABLES AND FIGURES

6.1 Figures

In Fig. S4, we show the performances of single and ensemble models.

In Fig. S5, we show the confusion matrices of the *Best_6_avg* and *Best_6_stack* models.

6.2 Tables

In Tab. S2 we compare the performance of our models on public datasets, with previous literature. The numbers in the table correspond to the data shown in Fig. 5 of the main text.

Model	ZooScan	Kaggle	WHOI
Best_6_avg (ours)	**0.898/0.915**	**0.947/0.937**	**0.961/0.961**
Best_6_stack (ours)	0.891/0.911	0.943/0.934	0.958/0.958
SFFS [9] (11 classifiers)	0.885/0.900	0.942/0.927	0.958/0.958
WS [9] (11 classifiers)	0.888/0.902	0.942/0.927	0.958/0.958
Fus_2R+Fus_1R [10] (24 classifiers)	n.a./0.897	n.a./0.926	n.a./0.953
Fus_PR+Fus_2R +Fus_1R [10] (43 classifiers)	n.a./0.896	n.a./0.926	n.a./0.953
NLMKL [11] (3 kernels)	n.a./0.894	n.a./0.846	n.a./0.900

Table S2. Performances Accuracy/F1-score of our *Best_6_avg* and *Best_6_stack* models on the publicly available datasets (ZooScan, Kaggle, WHOI), and comparison with previous results from literature. Different ways of creating ensembles are identified with the keywords SFFS (Sequential Forward Floating Selection - a feature selection method used to select models), WS (Weighed Selection - a stacking method that maximizes the performance while minimizing the number of classifiers) and Fus (fusion of diverse architectures and preprocessing) indicate different ways of creating ensembles [10, 9]. The last line stands for non-linear multi kernel learning (NLMKL), where an optimal non-linear combination of multiple kernels (Gaussian, Polynomial and Linear) is learnt to combine multiple extracted plankton features.

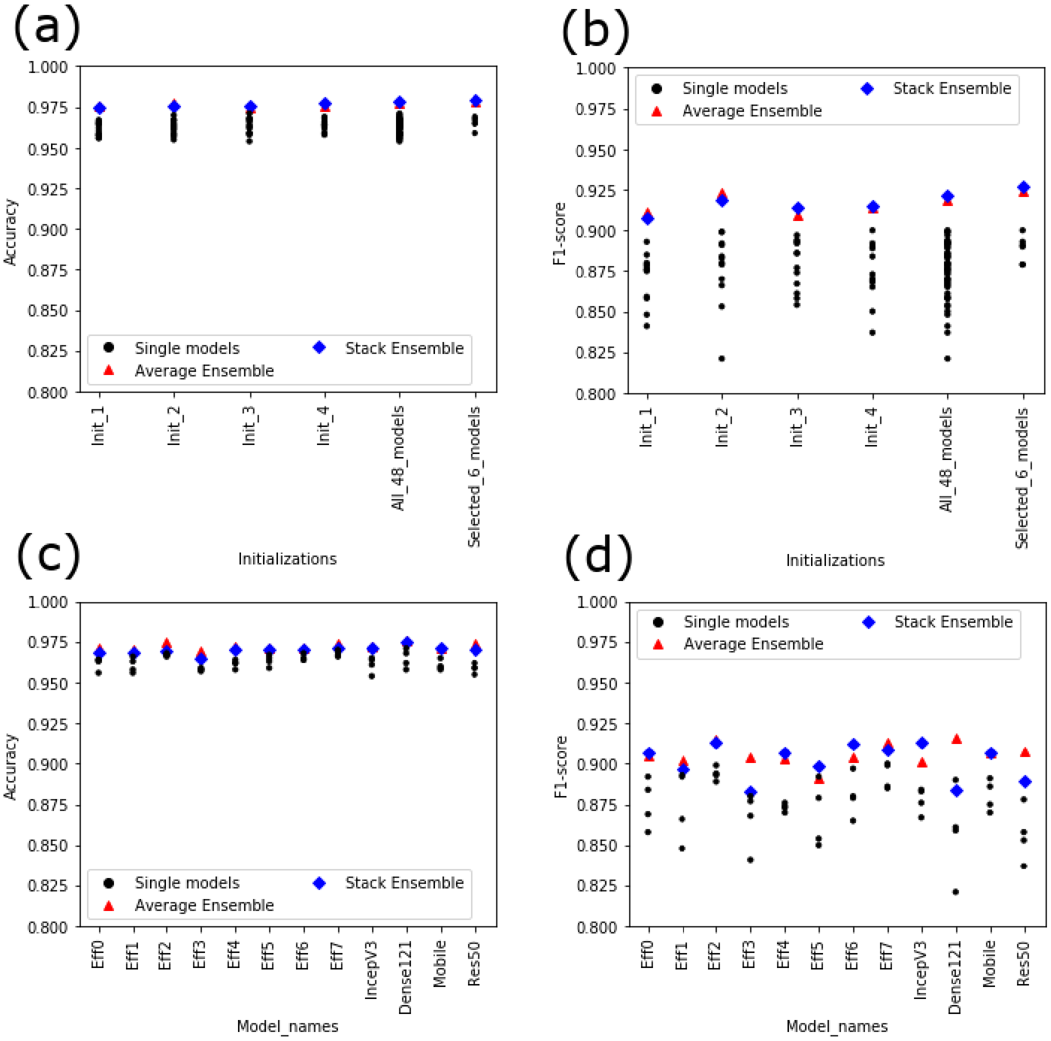

Figure S4. Performance of single and ensemble models (same data as Tab. 1). The solid circles are the single model performances.The red triangles represent the performance of average ensemble models, whereas the blue diamonds represent the performance of stacking ensemble models. **(a)**: The first four columns show the test accuracy of each initial condition across different models (this corresponds to going along a column in Tab. 1). The fifth column shows the performances of all 48 image models, and the sixth is restricted to the models constituting *Best_6_avg* and *Best_6_stack* . In all cases we show the result of ensembling over these models. **(b):** Same, but for the F1-score. **(c):** For each model, we show the test accuracy of the four chosen initial conditions, and of ensembling through them (this corresponds to going along a row in Tab. 1). **(d):** Same, for the F1-score. The readers can refer to the table 1 for the values of each single points on this figure

In Tab. S3 we show the amount of wallclock time required to train our models on an NVIDIA GeForce RTX 2080 Ti GPU.

REFERENCES

[1]Lucía Zarauz and Xabier Irigoien. Effects of Lugol's fixation on the size structure of natural nano–microplankton samples, analyzed by means of an automatic counting method. *Journal of Plankton Research*, 30(11):1297–1303, 08 2008.

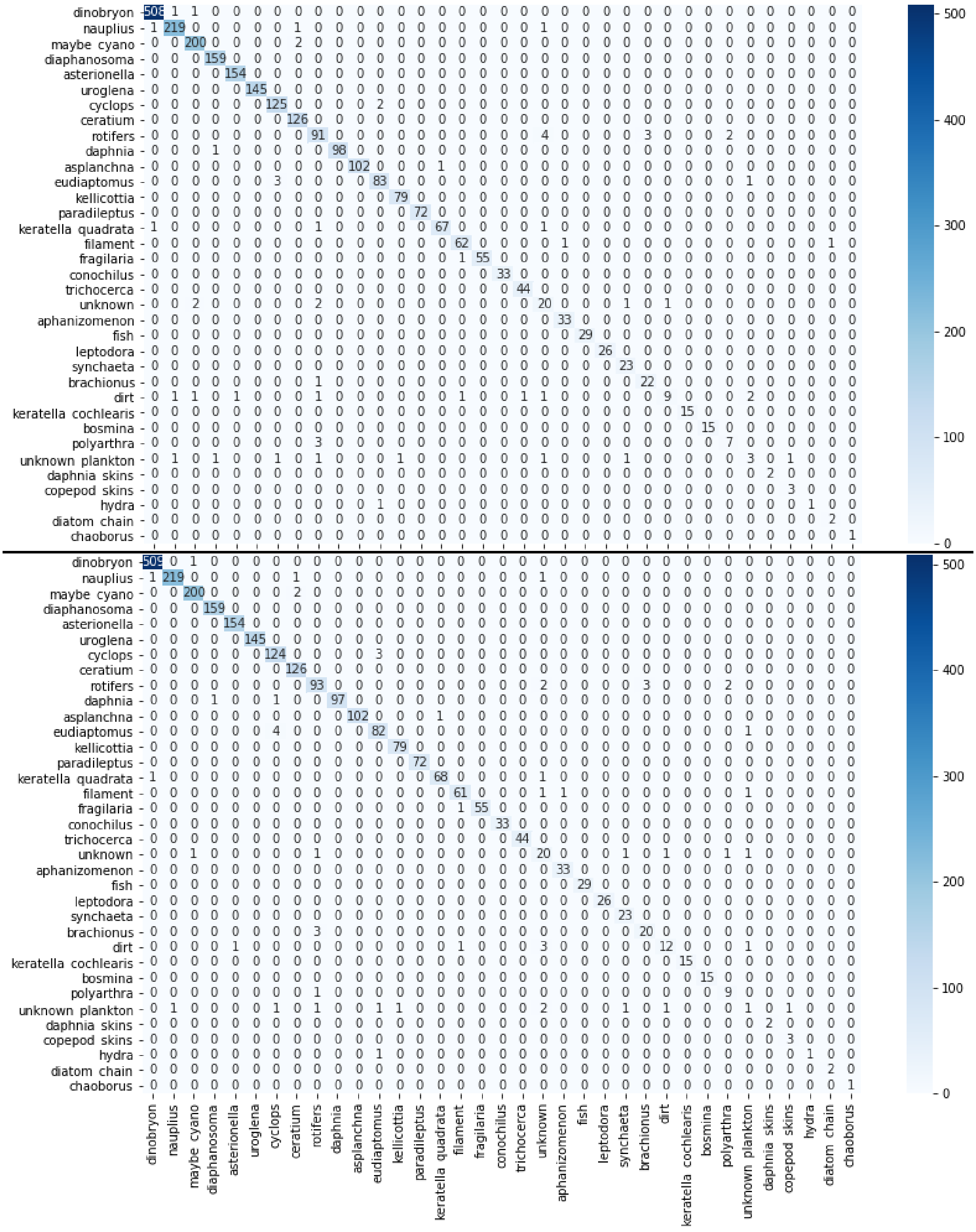

Figure S5. Confusion matrix of *Best_6_avg* (top) and *Best_6_stack* (bottom) Image models on test set sorted by overall abundance.

[2]T. Kerr, J. R. Clark, E. S. Fileman, C. E. Widdicombe, and N. Pugeault. Collaborative deep learning models to handle class imbalance in flowcam plankton imagery. *IEEE Access*, 8:170013–170032, 2020.

[3]Tom O'Malley, Elie Bursztein, James Long, François Chollet, Haifeng Jin, Luca Invernizzi, et al. Keras Tuner. `https://github.com/keras-team/keras-tuner`, 2019.

Model	Training time [hours]	Bayesian search [hours]
Eff0	3.8	14.8
Eff1	3.8	15.3
Eff2	3.0	15.6
Eff3	3.7	15.1
Eff4	3.5	14.7
Eff5	4.5	14.9
Eff6	5.5	15.3
Eff7	7.9	15.9
Mobile	3.2	14.7
Dense121	3.7	15.0
IncepV3	3.0	15.5
Res50	3.2	15.0

Table S3. Training times of our image models on an NVIDIA GeForce RTX 2080 Ti GPU. The second column represents the time to train a model across all phases of training, for a single choice of initial conditions and hyperparameters. The third column depicts the time required to choose the best hyperparameters.

[4]Justin M. Johnson and Taghi M. Khoshgoftaar. Survey on deep learning with class imbalance. *Journal of Big Data*, 6(1):27, Mar 2019.

[5]Stéphane d'Ascoli, Maria Refinetti, Giulio Biroli, and Florent Krzakala. Double trouble in double descent: Bias and variance (s) in the lazy regime. In *International Conference on Machine Learning*, pages 2280–2290. PMLR, 2020.

[6]Mikhail Belkin, Daniel Hsu, Siyuan Ma, and Soumik Mandal. Reconciling modern machine-learning practice and the classical bias–variance trade-off. *Proceedings of the National Academy of Sciences*, 116(32):15849–15854, 2019.

[7]Preetum Nakkiran, Gal Kaplun, Yamini Bansal, Tristan Yang, Boaz Barak, and Ilya Sutskever. Deep double descent: Where bigger models and more data hurt. *arXiv:1912.02292*, 2019.

[8]Mario Geiger, Arthur Jacot, Stefano Spigler, Franck Gabriel, Levent Sagun, Stéphane d'Ascoli, Giulio Biroli, Clément Hongler, and Matthieu Wyart. Scaling description of generalization with number of parameters in deep learning. *Journal of Statistical Mechanics: Theory and Experiment*, 2020(2):023401, 2020.

[9]Alessandra Lumini, Loris Nanni, and Gianluca Maguolo. Deep learning for plankton and coral classification. *Applied Computing and Informatics*, 2020.

[10]Alessandra Lumini and Loris Nanni. Deep learning and transfer learning features for plankton classification. *Ecological Informatics*, 51:33–43, 2019.

[11]Haiyong Zheng, Ruchen Wang, Zhibin Yu, Nan Wang, Zhaorui Gu, and Bing Zheng. Automatic plankton image classification combining multiple view features via multiple kernel learning. *BMC Bioinformatics*, 18(16):570, Dec 2017.
